# Comparative Analysis of Bone Resection Volume and Lateral Overhang in Four Closed-Wedge High Tibial Osteotomy Techniques—A 3D-CT Computational Simulation Study of Eleven Knees

**DOI:** 10.3390/jcm14207291

**Published:** 2025-10-15

**Authors:** Seok Jin Jung, Kyoung Won Park, Seung Joon Rhee, Young Woong Jang, Seong Jin Kim

**Affiliations:** 1 Department of Orthopaedic Surgery, Biomedical Research Institute, Pusan National University Hospital, Busan 49241, Republic of Korea; jsj4904@gmail.com (S.J.J.); pkw1533@gmail.com (K.W.P.); 2 College of Medicine, Pusan National University, Yangsan 50612, Republic of Korea; 3 Department of Molecular Medicine, Scripps Research, La Jolla, San Diego, CA 92037, USA; 4 Shiley Center for Orthopaedic Research and Education at Scripps Clinic, Scripps Health, La Jolla, CA 92037, USA; 5 Research and Development Center, Corentec Co., Ltd., Seoul 31056, Republic of Korea

**Keywords:** knee, osteotomy, tibia, osteoarthritis, realignment

## Abstract

**Purpose**: This study aimed to quantitatively compare the resected bony wedge volume and evaluate discrepancies in the non-overlapping lateral osteotomy surface areas among four closed-wedge high tibial osteotomy (CWHTO) techniques. **Materials and Methods**: Eleven knees from 10 patients who underwent high tibial osteotomy at our hospital (2016–2023) were analyzed using preoperative three-dimensional computed tomography. Representative cases were selected based on sex, the presence of proximal tibia vara, and a high joint line convergence angle. A subgroup analysis was then conducted. Surgical simulations were performed on reconstructed bone models using four different CWHTO techniques (conventional, oblique, hybrid 2:1, and hybrid 3:1) at three target angles (12°, 15°, and 18°). Osteotomy surface area and bony wedge volume were calculated and compared. **Results**: Distal osteotomy surface areas for the oblique, hybrid 1, and hybrid 2 techniques were 91%, 83%, and 72% of the conventional technique, respectively. Resected bony wedge volumes were 86%, 52%, and 38% of the conventional technique, respectively. Volumes decreased in the order of conventional, oblique, hybrid 3:1, and hybrid 2:1. Hybrid techniques showed significantly smaller resection volumes than the conventional and oblique techniques. The non-overlapping lateral osteotomy surface areas for oblique, hybrid 1, and hybrid 2 were 41% (lateral), 22% (medial), and 22% (medial) of the conventional technique, respectively. Only the conventional technique showed a statistically significant difference. **Conclusions**: Hybrid CWHTO techniques resulted in less bony wedge resection and fewer non-overlapping osteotomy surfaces compared with conventional and oblique techniques. Hybrid CWHTO may offer potential advantages in bone stock preservation and reduced lateral overhanging area.

## 1. Introduction

High tibial osteotomy (HTO) is a well-established surgical treatment for medial unicompartmental osteoarthritis and varus deformities of the knee joint. Since its introduction by Coventry MB [1], HTO has undergone continuous refinement to improve clinical outcomes. The two primary surgical techniques, classified based on osteotomy location and method, are medial open-wedge high tibial osteotomy (OWHTO) and lateral closed-wedge high tibial osteotomy (CWHTO) [2,3,4].

OWHTO offers the advantage of relatively easier intraoperative adjustment of the correction angle [3,5]. However, this technique carries risks such as nonunion or delayed union at the osteotomy site, frequently associated with hinge fractures [6,7]. In addition, it may unintentionally alter the posterior tibial slope and patellar height, potentially affecting postoperative knee biomechanics. Conversely, CWHTO facilitates earlier osteotomy site union and postoperative weight-bearing but has potential drawbacks, including lateral cortical bone contour distortion, peroneal nerve injury, and difficulty in compensating for over- or under-correction [8]. Despite the widespread adoption of OWHTO due to its relatively simple procedure and intraoperative flexibility for angle correction, CWHTO remains a viable alternative due to its distinct advantages [9].

CWHTO can be further categorized into three surgical techniques: conventional, oblique, and hybrid. The osteotomy method varies based on the hinge point’s location and the osteotomy line’s orientation. These variations influence the volume of bone resected and the configuration of the lateral cortex following osteotomy closure, even when the same correction angle is targeted. While individual advantages and disadvantages of each technique are not clearly delineated, postoperative changes in the lateral tibial configuration may influence fixation method selection. Notably, oblique and hybrid CWHTO techniques better accommodate locking plates compared to staples. Furthermore, bone loss may impact the feasibility of future conversion to total knee arthroplasty, and the lateral overhang area can alter proximal tibia morphology, potentially influencing the choice of fixation method.

We sought to determine the extent of differences in the volume of bone removed from the osteotomy gap and the step-off between opposing osteotomy surfaces based on the surgical technique used. We hypothesized that, compared with conventional CWHTO, oblique and hybrid CWHTO techniques would result in a progressive reduction in both bone volume resected and lateral non-overlapping area. Therefore, this study aimed to quantitatively compare and analyze the volume of bony wedge resected by simulating CWHTO using bone models for each technique (conventional CWHTO, oblique CWHTO, hybrid CWHTO). Additionally, we aimed to assess discrepancies in the non-overlapping lateral osteotomy surface areas among these techniques.

## 2. Materials and Methods

This study included 11 knees from 10 patients who underwent proximal tibial osteotomy for medial compartment osteoarthritis with varus deformity at our hospital between 2016 and 2023. These patients were selected for computed tomography (CT) analysis based on demographic and anatomical characteristics, including sex, age, proximal tibia vara, and a high joint line convergence angle (JLCA), ensuring representation of the general population eligible for HTO. Preoperative three-dimensional CT (3D-CT) data from these patients were analyzed.

Radiologic assessments of preoperative parameters were independently performed by two board-certified orthopedic surgeons. Measurements included hip-knee-ankle axis (HKA), medial proximal tibia angle (MPTA), JLCA, and the width of the proximal tibia on standing anteroposterior plain radiographs. 3D-CT (Revolution^®^, GE HealthCare Technologies Inc., Chicago, IL, USA) was performed with the patient in the supine position, ensuring that the leg was supported at the ankle to prevent lower limb external rotation. The patella was centered in the coronal plane to align the leg’s rotational axis appropriately. Axial images were taken with 2.5 mm slices from the hip joint to the ankle joint and extracted in the DICOM file format.

**Figure 1:**
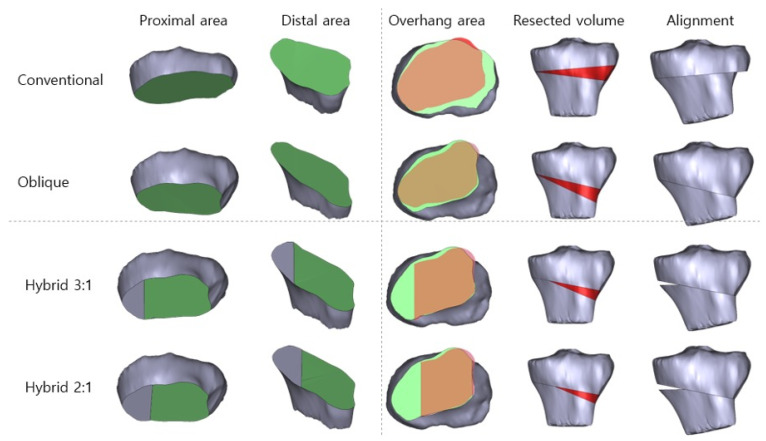
Schematic illustration of the surgical simulation for each closed-wedge high tibial osteotomy (CWHTO) technique. The overhang area refers to the non-overlapping region resulting from osteotomy closure. it is highlighted in light green.

The DICOM files were imported into Mimics (version 16.0, Materialize, Leuven, Belgium) and converted into the stereolithography (STL) format. The extracted data were then converted to the Standard for the Exchange of Product Data (STEP) format using ANSYS SpaceClaim (Version 2023 R2, ANSYS Inc., Canonsburg, PA, USA) to perform virtual CWHTO. Subsequently, surgical simulations were performed for four CWHTO techniques (conventional, oblique, hybrid 2:1, and hybrid 3:1) at three target angles (12°, 15°, and 18°) using SolidWorks (Version 2022, Dassault Systèmes, Vélizy-Villacoublay, France). Three target angles were chosen as representative of mild, moderate, and higher corrections commonly applied in medial compartment osteoarthritis. The saw-cut thickness was assumed to be 0 mm. The postsurgical simulation models were aligned in 3-matic (Version 8.0, Materialize, Leuven, Belgium) to measure the osteotomy surface areas and resected bone volume (Figure 1).

### 2.1. Surgical Simulation

Four CWHTO techniques—conventional [10], oblique [11], hybrid 1 [12], and hybrid 2 [13]—were simulated based on the hinge point position and osteotomy line orientation. In conventional CWHTO, the proximal osteotomy was performed parallel to the joint line, 10 mm distal to the proximal tibial joint surface, while the distal osteotomy extended from a distal location on the lateral cortex to the medial endpoint of the proximal osteotomy line, forming the desired correction angle [10]. In oblique and hybrid CWHTO, the proximal osteotomy connected a point 35 mm distal to the lateral tibial joint line with a point 15 mm distal to the medial tibial joint line. The hinge point in oblique CWHTO was located at the medial cortex, whereas in the hybrid technique, it was positioned at 3:1 (hybrid 1) and 2:1 (hybrid 2) from the medial cortex along the proximal osteotomy line. The distal osteotomy extended from the lateral cortex to the designated hinge point to form the desired correction angle (Figure 1).

### 2.2. Statistical Analysis

Continuous variables were presented as mean ± standard deviations or median (interquartile range) and compared using two-sample Student’s *t*-tests or the Mann–Whitney U tests. Categorical variables were analyzed using Pearson’s χ^2^ or Fisher’s exact tests. Statistical significance was set at *p* < 0.05. All statistical analyses were performed using SPSS (Version 27.0; IBM Corp., Armonk, NY, USA)

The study was conducted in accordance with the Declaration of Helsinki, and the protocol was approved by the Institutional Review Board of Pusan National University Hospital (2410-017-144) on 30 October 2024. As the study posed minimal risk to the participants, a waiver of written informed consent was granted.

## 3. Results

A total of eleven knees from 10 patients (five men and five women) with an average age of 58 years (range: 51–66 years) were included in the study. The mean preoperative proximal tibia width was 70.8 ± 6.2 mm (range: 60.4–80.7 mm), HKA was 10.5° (range: 5.1–16.5°), MPTA was 82.1° (range: 75.9–87.7°), and JLCA was 4.4° (range: 0.1–10.1°).

### 3.1. Proximal Osteotomy Surface Area

The proximal osteotomy surface areas measured 2370 ± 546 mm^2^, 1964 ± 454 mm^2^, 1962 ± 484 mm^2^, and 1962 ± 484 mm^2^ for the conventional, oblique, hybrid 1, and hybrid 2 techniques, respectively. The proximal osteotomy surface area was larger with the conventional technique than with the other three techniques, which had nearly identical proximal areas (Table 1).

### 3.2. Distal Osteotomy Surface Area

**Figure 2:**
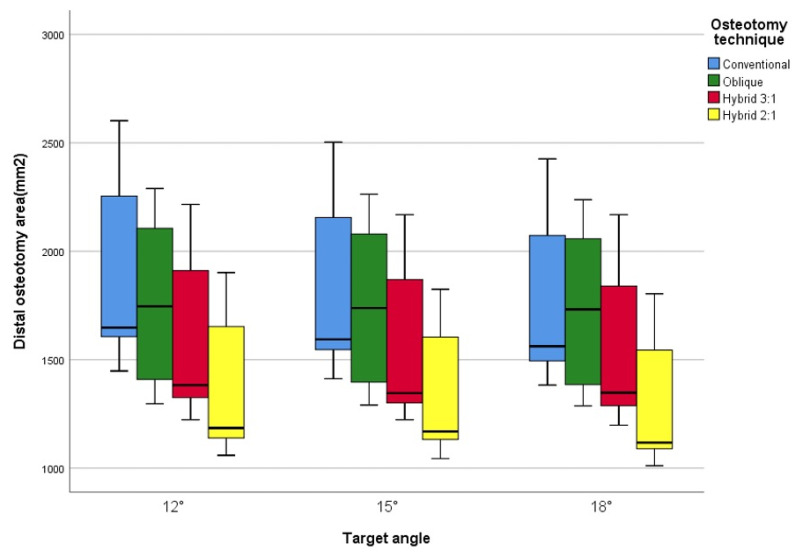
Distal osteotomy surface area values categorized by technique and target angles.

At a 12° correction angle, the distal osteotomy surface areas were 1962 ± 417 mm^2^, 1741 ± 375 mm^2^, 1595 ± 343 mm^2^, and 1382 ± 364 mm^2^ for the conventional, oblique, hybrid 1, and hybrid 2 techniques, respectively. Compared with the conventional technique, the oblique, hybrid 1, and hybrid 2 techniques yielded surface areas corresponding to approximately 88%, 81%, and 70%, respectively. A similar trend was observed at correction angles of 15° and 18°, as shown in Table 1 and Figure 2.

### 3.3. Non-Overlapping Osteotomy Surface Areas

**Figure 3:**
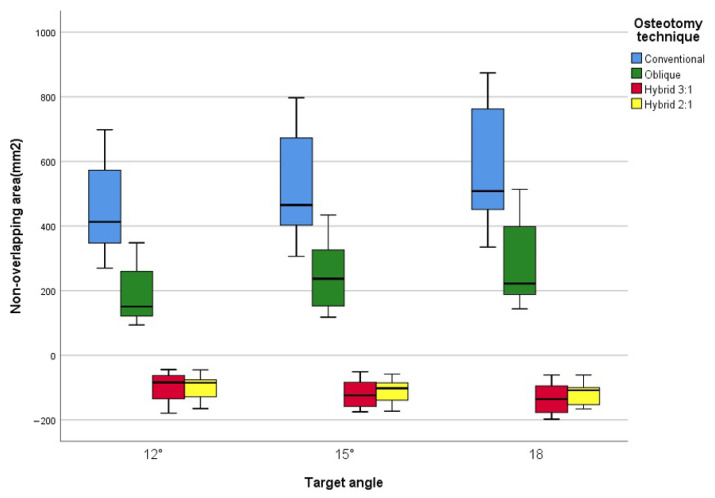
In the hybrid technique, the non-overlapping area is shown as a negative value due to the medial opening area.

At a 12° correction angle, the non-overlapping areas of the osteotomy surface were 458 ± 153 mm^2^ for the conventional technique, 190 ± 88 mm^2^ (lateral) for the oblique technique, 101 ± 46 mm^2^ (medial) for hybrid 1, and 102 ± 39 mm^2^ (medial) for hybrid 2. These values corresponded to approximately 41%, 22%, and 22% of the non-overlapping surface area observed with the conventional technique for the oblique, hybrid 1, and hybrid 2 techniques, respectively. A similar trend was observed at correction angles of 15° and 18°, as shown in Table 2 and Figure 3. In the hybrid techniques, non-overlapping areas were located medially due to the lateral cortex being seamlessly aligned during osteotomy gap closure, as illustrated in Figure 1. The conventional technique exhibited a statistically significant difference compared to the other three techniques, but no significant differences were observed among the oblique, hybrid 1, and hybrid 2 techniques (Table 2).

### 3.4. Volume of Resected Bony Wedge

**Figure 4:**
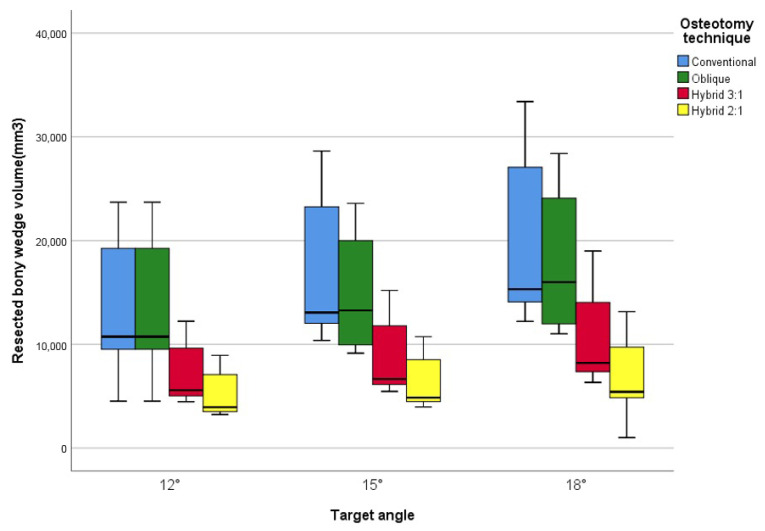
Lateral resected bony wedge volume categorized by technique and target angles.

At a 12° correction angle, the volume of the resected bony wedge was 13,558 ± 6075 mm^3^ for the conventional technique, 11,742 ± 4433 mm^3^ for the oblique technique, 7130 ± 2753 mm^3^ for hybrid 1, and 5281 ± 2253 mm^3^ for hybrid 2. These values corresponded to approximately 86%, 52%, and 38% of the non-overlapping surface area observed with the conventional technique for the oblique, hybrid 1, and hybrid 2 techniques, respectively. A similar trend was observed at correction angles of 15° and 18°, as shown in Table 3 and Figure 4. There were no statistically significant differences between the conventional and oblique techniques, or between the hybrid 3:1 and 2:1 techniques. However, both hybrid techniques (2:1 and 3:1) showed statistically significant differences compared with the conventional and oblique techniques (Table 3).

### 3.5. Subgroup Analysis

Further analysis of the primary results was performed by categorizing patients based on sex, proximal tibial varus angle (mMPTA < 83°), and high JLCA (JLCA > 7°). Male patients showed a significantly lower MPTA and wider tibia than that of female patients, likely due to their generally larger bone size. There were no significant differences in HKA, JLCA, or tibial width between the proximal tibia vara and non-vara groups. The HKA angle was greater in the high JLCA group compared to the normal JLCA group. Based on these criteria, the osteotomy surface area, resected bony wedge volume, and non-overlapping areas were reviewed. Significant differences were observed between the male and female groups, which were attributed to sex differences in bone size. However, no significant differences were found in the high JLCA group (Table 4 and Table 5).

## 4. Discussion

The most important finding of this study was that, among the various methods of lateral CWHTO, the hybrid techniques resulted in relatively smaller resected bony wedge volumes and smaller non-overlapping lateral osteotomy surface areas. This study provides objective data to quantitatively compare resected bony wedge volumes and non-overlapping lateral areas, which were previously understood only intuitively.

There are several well-known disadvantages associated with the conventional lateral CWTHO technique. The primary drawbacks include loss of bone stock, shortening of limb length, distortion of proximal tibial anatomy, and mechanical instability. In conventional CWHTO, a proximal osteotomy is performed parallel to the proximal tibial joint line near the knee joint, and a distal osteotomy is performed obliquely to the proximal osteotomy line. This method generates two highly mismatched osteotomy planes within the abruptly flared zone of the proximal tibial metaphysis, leading to unavoidable bone stock loss and significant postoperative lateral tibial anatomical distortion. Excessive bone loss contributes to limb shortening, whereas lateral cortical step-off compromises the overall stability of the construct by layering brittle cancellous bone over the sharp cortical bone wall [13]. During angular correction, plastic deformation occurs in the medial cortical hinge, and microfractures develop in this region as the osteotomy gap is closed. As the required correction angle increases, the risk of complete hinge fracture also rises [14]. In contrast, hybrid lateral CWHTO positions the hinge approximately 2:1 to 3:1 of the way into the medial cortex, effectively creating a controlled osteotomy gap rather than inducing a fracture. The medial gap created in hybrid HTO is minimal—approximately one-ninth to one-sixteenth the volume of the lateral bony wedge—and typically undergoes spontaneous bone healing. In this technique, the lateral plate functions biomechanically as a tension band, providing stable fixation and reducing the risk of fixation failure.

To address the limitations of conventional CWHTO, alternative surgical methods have been developed. The oblique CWHTO technique differs from the conventional approach by placing the first osteotomy line obliquely, which reduces lateral cortical step-off and enhances the fit for lateral locking plate fixation [11]. The hybrid CWHTO technique was introduced to achieve large angular corrections with relatively less bone stock loss and reduced anatomical distortion while retaining the primary advantage of CWHTO—faster osteotomy site union. These benefits facilitate early postoperative rehabilitation and full weight-bearing at an earlier stage [13,15,16].

In our study, the distal osteotomy surface area decreased in the following order: conventional, oblique, hybrid 1, and hybrid 2 and decreased with increasing correction angles of 12°, 15°, and 18°. The uncovered proximal osteotomy surface, represented by the non-overlapping area after osteotomy gap closure, increased with larger correction angles due to the decreasing distal osteotomy surface relative to the proximal osteotomy surface. The incremental increase in non-overlapping areas along with greater correction angles, was relatively larger in conventional and oblique osteotomies (2.3–2.9%) but smaller in hybrid HTOs (0.4–1.0%). However, the impact of changing osteotomy methods at the same correction angle was more substantial, with a 10% decrease in non-overlapping area between conventional and oblique osteotomies and a 4.5–7.0% decrease between oblique and hybrid osteotomies. The difference between conventional and hybrid osteotomies was 14–18%, which is considered significant. Additionally, the location of the non-overlapping area differed; in the hybrid HTO, this area was generated on the medial side, which did not affect the lateral structural integrity. This aspect makes hybrid HTO advantageous, as it results in less distortion of lateral proximal tibial anatomy, allowing for stable locking plate fixation and a durable cortex-on-cortex construct.

Bone stock loss, expressed as the volume of the resected bony wedge, was greatest in conventional CWHTO and decreased sequentially in oblique, hybrid 1, and hybrid 2 techniques. The hybrid HTO techniques showed a drastic reduction in resected volume, with an 18° correction in the hybrid group requiring less bony wedge removal than a 12° correction in the conventional or oblique groups. The substantial bone loss associated with conventional and oblique CWHTO may be associated with lower limb shortening and reduced proximal tibial containment, which could hypothetically affect subsequent conversion to total knee arthroplasty [17].

This study has several limitations. First, the sample size was relatively small for this type of research. To mitigate this limitation, we adjusted for variables such as sex ratio and proximal tibial morphology to better reflect distributions observed in the general population, thereby enhancing clinical relevance. Second, data were not obtained from real patients. Because direct measurement of these values was not feasible, we employed 3D rendering simulations to achieve highly precise measurements. Third, this study did not include clinical outcomes, which may limit the evaluation of each technique’s effectiveness in practice. Fourth, cut-line distance was not normalized to tibial width, which may influence scaling across morphologies. However, our intention was to compare average geometric effects between techniques under a consistent fixed-distance assumption.

## 5. Conclusions

Among the various lateral CWHTO techniques, hybrid HTO demonstrated the least non-overlapping osteotomy surface area and bony wedge resection volume compared to conventional and oblique CWHTO techniques. Hybrid CWHTO may offer advantages in preserving bone stock and reducing anatomical distortion, making it a preferable alternative to conventional CWHTO. At the same time, these findings are hypothesis-generating and require validation in biomechanical and clinical studies.

## Figures and Tables

**Table 1:** Proximal and distal osteotomy surface areas at different target angles. Conventional: conventional lateral closed-wedge osteotomy; Oblique: oblique lateral closed-wedged osteotomy; Hybrid 1: medial 3:1 hinged hybrid lateral closed-wedged osteotomy; Hybrid 2: medial 2:1 hinged hybrid lateral closed-wedge osteotomy.

	Conventional (mm^2^)	Oblique (mm^2^)	Hybrid 1 (mm^2^)	Hybrid 2 (mm^2^)
Proximal area Average	2370 ± 546	1964 ± 454	1962 ± 484	1962 ± 484
Distal area				
12°	1962 ± 417	1741 ± 375	1595 ± 343	1382 ± 364
15°	1844 ± 399	1725 ± 367	1558 ± 335	1336 ± 277
18°	1786 ± 379	1709 ± 360	1536 ± 335	1293 ± 277

**Table 2:** Non-overlapping lateral osteotomy surface areas at different target angles. Conventional: conventional lateral closed-wedge osteotomy; Oblique: oblique lateral closed-wedge osteotomy; Hybrid 1: medial 3:1 hinged hybrid lateral closed-wedge osteotomy; Hybrid 2: medial 2:1 hinged hybrid lateral closed-wedge osteotomy; Non-overlapping areas indicated in { } represent medially generated areas, which are located opposite to those in conventional and oblique HTO techniques. Values in ( ) indicate the ratio of the non-overlapping area relative to the proximal osteotomy area.

	Conventional (mm^2^)	Oblique (mm^2^)	Hybrid 1 (mm^2^)	Hybrid 2 (mm^2^)
12°	458 ± 153 (19.3%)	190 ± 88 (9.7%)	{101} ± 46 (5.1%)	{102} ± 39 (5.2%)
15°	527 ± 176 (22.2%)	242 ± 108 (12.3%)	{119} ± 42 (6.1%)	{112} ± 39 (5.7%)
18°	585 ± 196 (24.7%)	287 ± 130 (14.6%)	{134} ± 48 (6.8%)	{119} ± 34 (6.1%)

**Table 3:** Volumes of the resected bony wedge at different target angles. Values in ( ) represent the ratio of resected bone volume relative to the conventional closed-wedge high tibial osteotomy; Conventional: conventional lateral closed-wedge osteotomy; Oblique: oblique lateral closed-wedge osteotomy; Hybrid 1: medial 3:1 hinged hybrid lateral closed-wedge osteotomy; Hybrid 2: medial 2:1 hinged hybrid lateral closed-wedge osteotomy.

	Conventional (mm^3^)	Oblique (mm^3^)	Hybrid 1 (mm^3^)	Hybrid 2 (mm^3^)
12°	13,558 ± 6075 (100.0%)	11,742 ± 4433 (86.6%)	7130 ± 2753 (52.6%)	5281 ± 2253 (39.0%)
15°	17,055 ± 6620 (100.0%)	14,720 ± 5561 (86.3%)	8683 ± 3456 (50.9%)	6251 ± 2433 (36.7%)
18°	19,924 ± 7679 (100.0%)	17,719 ± 6696 (88.9%)	10,416 ± 4334 (52.3%)	6866 ± 3497 (34.5%)

**Table 4:** Patient demographics and comparison of anatomical characteristics. Proximal tibia vara was defined as a medial proximal tibia angle (MPTA) ≤ 83°, and high joint line convergence angle (JLCA) was defined as a knee joint line convergence angle of ≥7°. Asterisks indicate statistically significant difference.

N = 11	Sex			Proximal Tibia Vara			High JLCA		
	Men	Women	*p*	High MPTA	Low MPTA	*p*	High JLCA	Low JLCA	*p*
Sex	5	6		Men: 3 Women: 4	Men: 2 Women: 2		Men: 1 Women: 3	Men: 4 Women: 3	
HKA (°)	10.08 ± 1.8	10.83 ± 3.8	0.702	10.88 ± 3.1	9.8 ± 3.1	0.593	13.0 ± 2.4	9.01 ± 2.2	0.021 *
MPTA (°)	82.6 ± 3.4	88.85 ± 0.8	0.002 *	80.31 ± 2.5	85.22 ± 1.7	0.008 *	81.5 ± 3.8	82.4 ± 3.2	0.687
JLCA (°)	3.3 ± 2.1	5.35 ± 3.8	0.292	4.28 ± 3.6	4.62 ± 2.7	0.877	7.97 ± 1.7	2.37 ± 1.5	0.002 *
Tibia width (mm^2^)	75.8 ± 3.2	66.56 ± 4.9	0.006 *	71.54 ± 6.0	69.37 ± 7.3	0.609	69.65 ± 3.8	71.38 ± 7.5	0.683

**Table 5:** Surgical simulation results categorized by technique for comparative analysis. Conventional: conventional lateral closed-wedge osteotomy; Oblique: oblique lateral closed-wedge osteotomy; Hybrid 1: medial 3:1 hinged hybrid lateral closed-wedge osteotomy; Hybrid 2: medial 2:1 hinged hybrid lateral closed-wedge osteotomy. Asterisks indicate statistically significant difference.

Proximal Osteotomy Area (mm^2^)									
	Men	Women	*p*	High MPTA	Low MPTA	*p*	High JLCA	Low JLCA	*p*
Conventional	3028 ± 245.4	1915 ± 96.2	<0.05 *	2427 ± 587	2269 ± 495	0.409	2206 ± 419	2463 ± 596	0.198
Oblique	2569 ± 219.7	1678 ± 311.4	<0.05 *	1948 ± 462	1801 ± 363	0.322	1766 ± 423	2077 ± 440	0.056
Hybrid 1	2796 ± 398.2	1718 ± 89.7	<0.05 *	2148 ± 557	2069 ± 477	0.683	2027 ± 434	2172 ± 572	0.453
Hybrid 2	2554 ± 504	1625 ± 80	0.002 *	1977 ± 526	1937 ± 440	0.827	1894 ± 402	2001 ± 539	0.555
Distal osteotomy Area (mm^2^)									
	Men	Women	*p*	High MPTA	Low MPTA	*p*	High JLCA	Low JLCA	*p*
Conventional	2307 ± 192	1529 ± 79	<0.05 *	1893 ± 433	1764 ± 300	0.325	1712 ± 273	1923 ± 430	0.095
Oblique	2167 ± 87	1444 ± 168	<0.05 *	1770 ± 381	1645 ± 307	0.343	1541 ± 330	1829 ± 334	0.023 *
Hybrid 1	2004 ± 155	1295 ± 55	<0.05 *	1598 ± 355	1501 ± 277	0.391	1455 ± 268	1624 ± 348	0.158
Hybrid 2	1713 ± 129	1107 ± 51	<0.05 *	1372 ± 307	1275 ± 236	0.352	1234 ± 231	1395 ± 299	0.119
Non-overlapping area (mm^2^)									
	Men	Women	*p*	High MPTA	Low MPTA	*p*	High JLCA	Low JLCA	*p*
Conventional	821 ± 109	383 ± 73	<0.05 *	533 ± 177	504 ± 187	0.662	493 ± 162	539 ± 188	0.484
Oblique	358 ± 74	168 ± 54	<0.05 *	253 ± 117	214 ± 108	0.348	224 ± 100	248 ± 122	0.571
Hybrid 1	153 ± 37	97 ± 41	<0.05 *	129 ± 45	98 ± 44	0.065	2242 ± 3352	2586 ± 3943	0.801
Hybrid 2	138 ± 38	97 ± 29	0.002 *	122 ± 38	90 ± 26	0.015 *	117 ± 26	107 ± 42	0.479
Resected bony wedge volume (mm^3^)									
	Men	Women	*p*	High MPTA	Low MPTA	*p*	High JLCA	Low JLCA	*p*
Conventional	24,964 ± 5522	11,590 ± 2616	<0.05 *	17,671 ± 7850	15,400 ± 5630	0.386	15,264 ± 5272	17,749 ± 7962	0.291
Oblique	21,817 ± 4114	10,538 ± 2486	<0.05 *	15,553 ± 6379	13,279 ± 5198	0.302	12,707 ± 5241	11,726 ± 9823	0.711
Hybrid 1	13,426 ± 2958	6093 ± 1048	<0.05 *	9155 ± 4028	8021 ± 3136	0.408	5820 ± 4942	6730 ± 5931	0.657
Hybrid 2	9635 ± 1808	4089 ± 1023	<0.05 *	6605 ± 2862	5307 ± 2516	0.201	5205 ± 2456	6664 ± 2861	0.149

## Data Availability

The original contributions presented in this study are included in the article. Further inquiries can be directed to the corresponding author.
